# Survey Mode Effects on Valuation of Environmental Goods

**DOI:** 10.3390/ijerph8041222

**Published:** 2011-04-18

**Authors:** Jason Bell, Joel Huber, W. Kip Viscusi

**Affiliations:** 1 Fuqua School of Business, Duke University, Durham, NC 27708, USA; E-Mails: jbb@duke.edu (J.B.), jch8@mail.duke.edu (J.H.); 2 Vanderbilt Law School, Vanderbilt University, 131 21st Avenue South, Nashville, TN 37203, USA

**Keywords:** survey mode, environmental economics, internet surveys, stated preference, benefit-cost analysis, water quality

## Abstract

This article evaluates the effect of the choice of survey recruitment mode on the value of water quality in lakes, rivers, and streams. Four different modes are compared: bringing respondents to one central location after phone recruitment, mall intercepts in two states, national phone-mail survey, and an Internet survey with a national, probability-based panel. The modes differ in terms of the representativeness of the samples, non-response rates, sample selection effects, and consistency of responses. The article also shows that the estimated value of water quality can differ substantially depending on the survey mode. The national Internet panel has the most desirable properties with respect to performance on the four important survey dimensions of interest.

## 1. Introduction

The choice of survey recruitment mode has a potentially important influence on the measurement of the value of environmental goods based on survey responses. There are two principal dimensions of influence that we analyze in this article. First, the mode influences who chooses to respond to the survey, thus affecting the extent to which the responses reflect the valuations of the population of interest. Second, for the particular sample of respondents, the survey recruitment mode affects whether the survey elicits their preferences accurately. Thus, the mode alters how they respond to the survey questions and the valuations that are elicited. This article explores the valuation of a single environmental commodity using different survey recruitment modes.

There is a substantial literature regarding the effects of survey mode on responses, particularly due to the increasing difficulty over time of reaching potential survey respondents by phone, mail, and e-mail. Lindhjem and Navrud have an excellent discussion of research on survey mode effects [1]. This difficulty has led to the use of convenience samples, using both phone lists and opt-in Internet samples, samples drawn from mall intercept recruiting, and samples drawn from panels. Of great concern is whether the mode by which a survey is implemented affects response rates, results, and the demographic characteristics of the surveyed sample. For instance, research such as Dillman *et al.* has shown that questions presented visually can lead to different answers compared to questions presented aurally, particularly for questions using scales [2].

The nature of our computer-based interactive survey and cost considerations led us to explore a series of survey modes other than door-to-door in person interviews. Several previous studies have considered that survey mode and the evidence regarding face-to-face interviews is mixed. Surveys administered in the presence of an interviewer may not be the gold standard as this survey mode has been shown to influence responses due to a social desirability effect, or a conscious or subconscious tendency on the part of a respondent to give answers that might please the interviewer. Maguire found that respondents to a face-to-face interview were more likely to agree to participate in a survey about hypothetical charitable contribution than telephone respondents, and the amount of the contributions by those interviewed face-to-face were smaller on average than mail respondents among those who agreed to contribute [3]. Leggett *et al.* found that respondents interviewed face-to-face about the amount they would pay to visit a national park had values over 20% higher than those who self-administered the survey on paper [4]. Similar effects were found by Marta-Pedroso *et al.* in comparing face-to-face interviews with Internet responses to a survey about environmental preservation in Portugal [5]. These results are not unanimous, however, as Covey *et al.* reached similar results with face-to-face and Internet surveys on rail safety [6], while Lindhjem and Navrud did not find significant differences between face-to-face and Internet interviews drawn from the same panel [1]. The survey that is the focus of this research should minimize social desirability, as all of the surveys were self-administered either on disk using a computer program or via the Internet using a computer or other web-enabled device.

While those studies concentrate mainly on the differences in responses based upon how the survey is administered (in person, over the phone, on paper, or electronically), this research examines how values differ when all respondents take the survey the same way (electronically either on computer or over the Internet), but are recruited in different ways (by phone, in person at a mall, or electronically invited from an existing panel) and complete the survey at different locations (at home, at a designated location, or at the location where they were recruited). We find that the manner in which potential respondents are recruited, the likelihood of respondents to self-select into or out of the survey, the costs in time and effort imposed by the survey, and the diligence with which respondents complete the survey task each can affect the estimated value of the good.

The substantive focus of the survey is the valuation of improvements in inland water quality. In particular, how much do people value increases in the quality of lakes, rivers, and streams? The quality dimensions of interest include the recreational uses of swimming and fishing, and also include ecological benefits to plants, fish, and wildlife that are associated with clean water. People may value many of these benefit components regardless of whether they visit lakes and rivers. While ecological benefits are mostly associated with non-use, we found non-use values for all the water quality features. The estimated values combine use and non-use values of the features. As a consequence, attempts to elicit monetary values of water quality based on recreational visits to lakes and rivers cannot capture all the benefits associated with water quality. To obtain these values, some kind of survey approach that can elicit meaningful measures of water quality is essential. Because the survey structure we have designed involves an interactive computer-based valuation task, the survey mode must be able to both accommodate computer implementation and create a context in which a representative sample of respondents can give thoughtful responses to the valuation task.

To examine the influence of survey mode, we investigate the differences in responses to an interactive computer survey using four survey modes: central location, mall intercept, phone-mail, and Internet panel. These modes differ in terms of the manner of recruitment, the costs they impose on the respondent, and the environment in which the survey is administered. The range of survey modes examined here is not intended to be exhaustive. For example, we do not consider door-to-door surveys because of the increasingly high cost of obtaining a representative sample of respondents to an interactive computer survey based on door-to-door visits by a survey firm representative.

We find substantial differences across the four modes in terms of monetary values of water quality. Differences arise both because of who responds to the survey and how they respond. Our examination of the differences in demographic characteristics of participants highlights the effect of the survey mode on the selection of respondents into the sample pool. By analyzing the predicted environmental benefit values controlling for demographic mix we also can demonstrate that there is an important selectivity effect that biases the empirical estimates in the econometric model. Our review of the performance of the survey modes also indicates substantial differences in the rates of inconsistency in answering the survey questions, which is one measure of how the survey mode affects respondents’ ability to give meaningful answers to the valuation task.

We also consider several important objectives of a successful survey administration and examine how well each survey mode fostered those objectives. In particular, we conclude that a probability based Internet panel is best suited to the objective of tapping a representative sample of potential respondents. Internet panels mitigate the effects of respondents self selecting disproportionately into particular topics, such as the environment, in which they have strong interest; they limit the time and travel costs associated with completing the survey, and they enable the survey to be taken at home where the respondent is comfortable completing the survey. Phone-mail, central location, and mall intercept modes have favorable features with respect to some of these objectives and may be preferable if cost is a major concern, but are problematic with respect to one or more of survey evaluation criteria.

We begin by describing the survey instrument since the instrument will affect which survey modes are feasible and their relative merits in eliciting benefit values. We then examine the various modes used for fielding the survey. Following this discussion, we review the key dimensions on which surveys should be judged. Using these criteria, we analyze the valuation results obtained using each of the survey modes. Then follows an analysis of the extent of inconsistent responses in each survey mode, an important measure of the degree to which the survey mode helps or hinders the generation of accurate and useable individual valuations.

The representativeness of a survey sample can be assessed in two ways. First, for each survey mode it is possible to compare the sample characteristics with the national adult population. Second, since the Internet panel collects demographic information on all panelists, for that survey mode the presence of any sample selection effects can be estimated. We examine demographic effects in terms of the choice to participate in the Internet panel survey and compare those effects with the demographics of the other survey modes to reveal the extent to which each mode experiences sample selection effects. Our concluding discussion reviews the reasons that we believe that a nationally representative Internet-based panel drawn using a probability sample of the U.S. population is the most meaningful approach.

## 2. Survey Instrument

**Figure 1.**
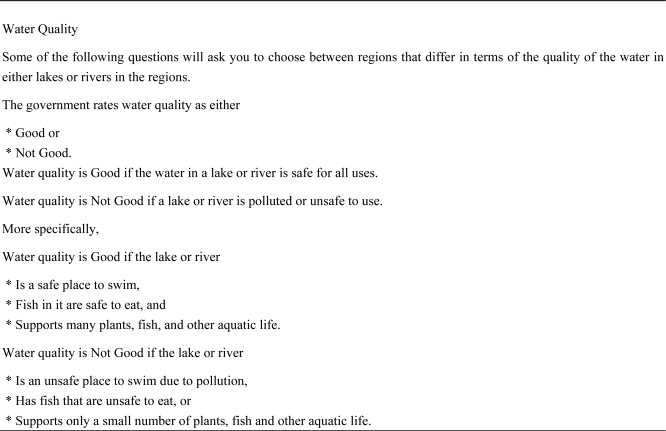
Text of water quality definition in survey.

The survey used in this analysis focuses on the value of water quality for inland water—lakes, rivers, and streams. Specifically, the survey elicited the monetary value of lake and river quality in a respondent’s region. These dimensions, shown in Figure 1, reflect the water quality dimensions used by the U.S. Environmental Protection Agency (EPA) for its National Water Quality Inventory, a measure of water quality conditions in the United States (this document can be found on the EPA website at http://water.epa.gov/lawsregs/guidance/cwa/305b/index.cfm).

The dimensions are whether fish caught in the lake or river were safe to eat, whether swimming in the water could make one ill, and whether the lake or river supported a healthy environment of plants, fish, and other aquatic life. The safety of water for drinking is explicitly excluded as a matter of concern since respondents are told that water treatment facilities address drinking water quality issues.

In order to avoid focusing on idiosyncratic aspects of the respondent’s region that could not be monitored and might affect responses in unpredictable ways, the survey asked respondents to think about a hypothetical move to one of two new regions that resembled their own region in terms of number of water bodies and general characteristics. The goal of this multiple-question set is to obtain a meaningful point valuation for each individual respondent for an unfamiliar, non-market good using a few relatively simple choices. The survey approach uses a series of iterative paired comparisons patterned after the approach pioneered by Viscusi, Magat, and Huber [7]. These are pairwise regional choices that differ on two dimensions: water quality and cost of living. For further information regarding the iterative choice method used in the survey instrument, see Magat, Huber, and Viscusi [8].

**Figure 2.**
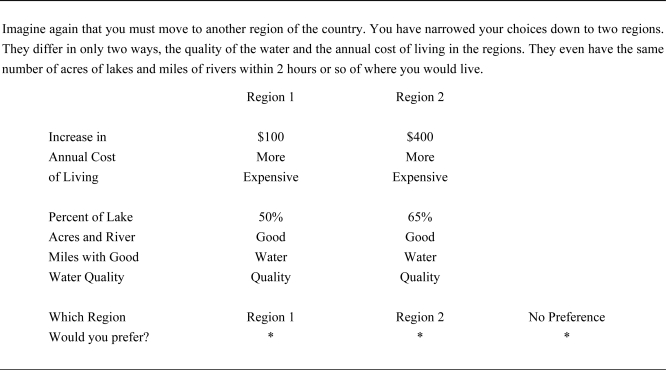
Text of water quality survey question.

Figure 2 shows the text of a representative question. Respondents first choose one of the two regions. Based on the individual response, the survey then alters the choice comparison to make the choices more equally valued, where the overall objective is to find the point of indifference between the two regions.

As part of this iteration process, subsequent questions either reduce the difference in water quality between the two regions or reduce the difference in cost of living between regions to estimate the point at which the respondent is indifferent between the presented options. Even if the respondent does not indicate strict indifference between the options, the survey generates a bounded value for the dollar value of improved water quality that lies between the tradeoff rates for the answers to the two sequential questions bracketing the last switch. There is only an upper or lower bound where the respondent reaches a corner of the iterated question set without ever switching. In those cases valuation is estimated econometrically using censored regression models.

**Figure 3.**
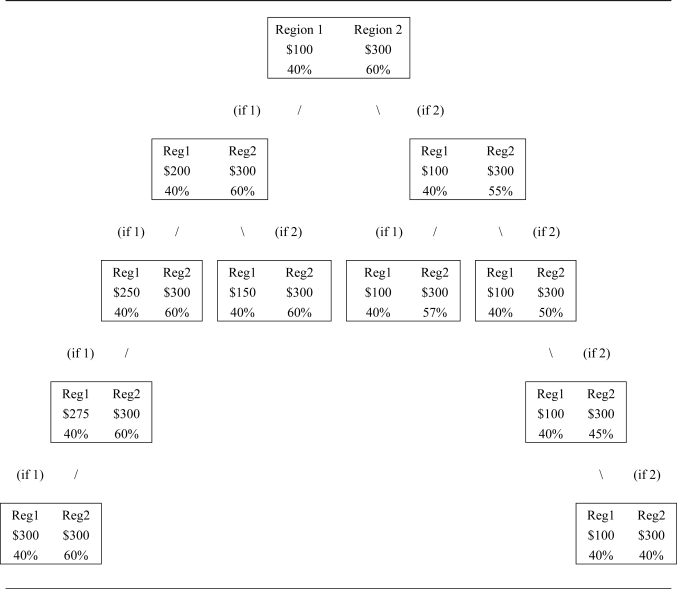
Survey decision tree.

Figure 3 shows a sample iteration tree for the question set. The starting point is a choice in which the respondent must pay a $200 premium for a 20% increase in water that is rated as being of Good quality, or $10 per 1% increase in water quality. Respondents who value water quality at more than this amount consider the succession of choices on the right side of the tree for which the regional cost difference remains unchanged but the difference in water quality rating is reduced. Respondents who indicate a lower valuation of water quality on the initial choice go down the left side of the tree in which the water quality difference remains unchanged and the regional cost difference narrows.

For those respondents who reached a corner solution we include other questions to test for the rationality of the choice. In the question following the final question iteration (the fourth question down the left or right side in Figure 3), the previously chosen option becomes dominated by the alternative. Respondents choosing the dominated option are informed that they have done so and are given the opportunity to alter their response. These inconsistent respondents who fail to alter their response either do not understand the process or are actively protesting it. The percent of inconsistent respondents provides an important measure differentiating the four survey modes.

## 3. Survey Modes

The survey was fielded fourteen times from August 1997 to October 2004. All of the surveys were restricted to those older than 18 years and were administered on computers. Table 1 provides information on the timing and implementation of the phone recruitment to a central location in North Carolina, the mall intercepts in North Carolina and Colorado, the national phone-mail, and the national Internet panel recruited by Knowledge Networks (KN). These efforts produced a total of 5,122 completed surveys, each of which can be used to generate an estimate of a respondent’s value of a one percentage point change in water quality, adjusted for inflation to 2004 dollars. This value and the demographic characteristics of each respondent serve as the principal basis of the analysis. We augment the examination of survey modes with additional measures such as the frequency of inconsistent responses.

The first survey mode that was used involved bringing respondents to a central location. This survey was administered by the marketing firm Johnston-Zabor and Associates in 1997 in Research Triangle Park, North Carolina. The survey firm recruited people by phone from a convenience sample of respondents that had completed surveys in the past. The survey firm asked people to visit a central location to complete the survey on a computer.

The second survey mode was a standard mall intercept survey administered in shopping malls in Cary and Charlotte, North Carolina, and Colorado Springs and Denver, Colorado in 1998 by the marketing firm Consumer Pulse. Representatives of the firm recruited mall shoppers to participate in the survey using computers at the mall location.

The phone-mail mode was also conducted by Consumer Pulse in late 1999 and mid 2000. Nationwide random digit dialing recruited the sample. After agreeing to participate, respondents received a disk by mail, which they used to complete the survey on their own computers. After doing so, they returned the completed survey disk by mail. Those without computers were offered additional compensation to use a neighbor’s computer or a computer available at a public location. Though over 75% of this sample used their home computer, 8% used a computer at work, and 13% used a friend’s computer. Only about 3.5% of respondents used a public location.

Knowledge Networks conducted the Internet panel mode between 2001 and 2004. This sample consisted of people previously recruited by nationwide random digit dialing to join a panel to take surveys online. KN invited a group of panel members to participate in our survey. Additional information on the characteristics of the KN panel can be found on the KN website at http://www.knowledgenetworks.com/knpanel/docs/KnowledgePanel(R)-Design-Summary-Description.pdf.

While the survey questions were similar throughout the modes, there were some differences. First, the starting cost and quality differences between regions presented to respondents differed both within the Internet panel administration as well as between survey modes. For instance, respondents in the Internet panel were presented starting cost-to-quality ratios between $5 per 1% quality difference and $30 per 1% quality difference. Thus, depending on which starting ratio the respondent received, the initial questions asked whether the respondent was willing to pay at a rate of $5 or $30 per 1% improvement in water quality. The central location mode had a starting tradeoff ratio of $4, and the phone-mail and mall intercept had a starting tradeoff ratio of $10. As described in Huber, Viscusi, and Bell, higher starting ratios can result in higher final valuations [9]. Accordingly, the influence of starting ratios on the respondent’s valuation is accounted for in the regression analysis.

Additionally, the survey modes differed in terms of the range over which the water quality differences spanned. The lowest, or baseline percentage of water rated of good quality, presented water quality rated good as ranging from 20% to 75% in the Internet panel and mall intercept, while the central location and phone-mail presented 50% as the baseline water quality. Previous research in Huber, Viscusi, and Bell found that these starting points influence values, with higher baseline quality leading to lower cost-quality tradeoff values as levels of water quality have a diminishing marginal value to respondents [9]. Even though the mean baseline quality was similar across modes, the potential influence of the starting level of water quality on valuations is also accounted for at the level of the individual in the regression analysis.

Finally, the phone-mail and Internet panel surveys contained a slightly larger question set. If respondents in those surveys continued to choose the option with higher cost and higher water quality, they were asked one additional question relative to the other modes before being presented with the dominated choice. Using Figure 1 to illustrate, the earlier surveys would have presented high-valuation respondents a 15% quality difference (65%–50%), then 10% then 5%, then 0%. The later surveys would have added a question with a 3% quality difference. The practical effect of this additional question, all else equal, should be fewer respondents whose values are censored at high values for the phone-mail and Internet panels because of the additional opportunity to switch choices of region, and those surveys could generate higher tradeoff rates for values at the censored point ($300/5% or $60 for earlier surveys and $300/3% or $100 for later surveys). Thus the censored regression takes account of these differences in the depth of iterative questions asked.

## 4. Sample Selection Differences among Survey Modes

Survey participation is a function of:

1. Ability of investigators to contact a potential respondent,
2. Interest of a potential respondent in the topic,
3. Total time and effort cost for a respondent to complete the survey, and
4. Ability of a respondent to be comfortable in the location where the survey is completed.

The most desirable mode will increase the performance on dimensions (1) and (4), will promote survey participation independent of dimension (2), and minimize the time and effort cost dimension (3).

We investigate how responses to survey questions vary among recruitment modes. All respondents answered the survey analyzed here electronically, either via the Internet or with a computer recording responses to a disk. Because of this similarity in administration, as well as the collection of personal characteristics of every respondent, the differences between survey responses can be measured largely on the basis of how and whether a respondent was recruited to participate and the relationship of each mode to the four factors listed above.

An objective of any survey is to get a representative sample of a target universe so as to obtain unbiased valuations of water quality. In our case, an objective was to have a sample representative of the adult U.S. population. There was, of course, no expectation that the results from a survey administered in a single region such as North Carolina or Colorado would reflect national preferences. These regional surveys served to explore how people would respond to the survey questions. The discussion below highlights some of the regional differences that arise. However, our main interest here is with potential selection biases in the four recruiting modes that limit the ability of the researcher to project the results to any target universe. The limitations based on selective regional coverage are not inherent shortcomings of the survey mode as one could, following the previous example, use mall intercepts throughout the country.

Differences between respondents and non-respondents are also of concern and have been evident in previous survey research. For instance, Rodes *et al.* found age, gender, urban/rural, and health related effects between early and late responders to a 1981 health-related survey in Spain using mail recruitment, with multiple telephone and in-person follow-ups for non-respondents [10].

Interest in the survey topic can be a significant determinant of survey participation. This has been previously noted as a factor in survey participation for mail surveys in Martin and Roberson and Sundstrom [11,12]. We also investigate whether differences in level of interest translate into value differences across recruiting modes. MacDonald *et al.* found that a non-panel Internet sample had much smaller response rates than a mail survey, and that the Internet respondents were wealthier, younger, and had better expertise on the survey subjects (farming and river recreation) despite having less experience in those subjects [13]. However, Olsen compared an Internet panel to a mail survey on protecting landscape from road encroachment and found that Internet respondents had a lower degree of estimation precision and reliability, despite a higher stated certainty and confidence in their choices [14].

In research using the KN Internet panel, Dickie *et al.* found that compared to results from a central location administration, Internet respondents had less knowledge of the subject (skin cancer), had more survey questions left unanswered, either rushed or took breaks during the survey, and failed the scope test whereby people should have higher valuations for greater delays in the onset of the skin cancer [15]. Our survey experience with the KN panel also included some respondents who completed the survey quickly or took long breaks, but we did not have difficulty with respondents failing pertinent scope tests in which greater increases in water quality should be valued more highly. This could be due to a series of questions used in our survey to explain the concepts and to engage the respondents about their own experiences with them, or the subject matter of this survey may have been less complicated than the skin cancer survey. We also found no significant effect of length of time in the panel on valuations, so multiple-survey fatigue does not appear to be a major concern. Unfortunately, problems with keeping respondents on task may be an inevitable trade-off where ensuring that respondents take the survey in a comfortable environment is a priority.

## 5. Demographic Differences among Modes

Because the survey modes differ in terms of their ability to reach the target population, one can expect differences in the demographic characteristics between survey modes. In each case we use the U.S. Census adult population as the reference point for determining the representativeness of the sample. Table 2 shows the portion of the sample that took the survey through KN probability based Internet panel. Overall, the sample characteristics closely match the demographics of the adult population in the United States. This matching is to some extent due to the fact that the demographics of the Internet panel are known before invitations are sent so that KN can draw a nationally representative sample of respondents for such studies.

Since a large majority of the full sample was drawn from this Internet panel, the full sample matches the U.S. adult population to a greater extent than might be expected considering the differences evident in the other modes. The close match is also due to the fact that potential respondents are already known to be willing to take surveys by their participation in the panel, making their participation more likely than it would be when the demographic effects of respondent interest in the survey topic are taken into account. This willingness could lead to other differences related to panel membership, such as whether the taking of multiple surveys affects answers to the next survey, discussed in Taylor *et al.* [16]. However, for respondents with data for tenure in the panel available, we found only slight positive correlations between tenure and inconsistency (0.04) as well as responses that had the lowest consistent value (0.04), and no correlation between tenure in the panel and their value for the good. Both significant correlations were at the 5% level. This subsample had 3,179 respondents with tenures ranging from zero to 60 months.

The other survey modes perform much less well in terms of matching the respondents to national population characteristics. It should be noted again that these modes had much smaller sample sizes than the Internet panel, so larger deviations should be expected as a matter of course. Even so, there were several statistically significant demographic differences from the adult U.S. population that are strongly related to the mode used. For instance, compared to the rest of the full sample which closely matched the U.S. adult population, the phone-mail sample included dramatically fewer respondents under 35 years of age (t = 4.89), comparing the subsample with the remainder of the data, as do the rest of the reported t-tests), almost no respondents with less than high school education (t = 3.61), twice as many college graduates or higher (t = 5.61), fewer minorities (t = 3.05), many more married respondents (t = 3.15), and few respondents with incomes below $15,000 (t = 2.92). These differences are as expected; older, married, wealthier, and more educated respondents should be more likely to be contacted, to receive the mailed survey materials, to complete the survey, and to mail it back.

Unfortunately, the phone-mail sample had a much higher frequency of respondents who have visited a lake or river in the last year than other modes (t = 4.24), indicating that there was substantial self selection with respect to valuation. The bottom of Table 2 shows that over 90% of respondents in the phone-mail sample indicated they had visited lakes or rivers in the past year as compared to 68% with the other survey modes. This difference in visitation percentages may arise from self selection, where potential applicants decided whether to participate based on the subject matter of the survey. This bias can be limited but not eliminated by including the regression coefficient for whether the respondent visited lakes or rivers.

This self-selection effect is also problematic for the central location sample, also a mode where the sample was recruited by telephone. As with the phone-mail mode, that sample had a high 89% (t = 4.53) of respondents reporting a visit to a lake or river in the last year. The central location sample had disproportionately fewer respondents in the youngest group of 18–24 (t = 1.95), though it had more of the next age group of 25–34 (t = 1.92). The central location respondents were much less likely to be Hispanic (t = 2.82), were wealthier, and were more educated than the other modes (t = 12.60 for the highest education level). Presumably, some of these differences are due to the same factors as phone-mail, but are also due to the location of the survey, Research Triangle Park in North Carolina. That area has a higher portion of college educated professionals, especially among those reachable by phone and who were able to easily make the trip to the survey location. Over 75% of the central location respondents had a college degree, even higher than the 47% with that level of education in that region overall (as reported at http://www.researchtriangle.org/uploads/pdfs/RTRP_Region.pdf).

The mall intercept sample had expected differences from national demographics. That sample is skewed to be much younger than the other modes (t = 11.10 and 2.59 for the two youngest age categories), has the highest rate of participation among black respondents (though not large enough to be statistically significant in a t-test), and is the only mode with a majority of unmarried respondents (t = 5.93). The mall intercept sample is also less educated (t = 3.18) and less wealthy (t = 4.40 and 6.51 for the two lowest income groups) than either mode first contacted by phone. These characteristics are not surprising, as respondents were recruited from the subsample of people visiting a shopping mall with enough time to interrupt their shopping trip to take 25 minutes to complete the survey. However, this sample seems to suffer much less from the self selection based on interest in the topic of lakes and rivers, as 75% of respondents report having visited a lake or river in the last year (t = 1.97). This percentage is still higher than the 68% among the Internet panel sample, where panelists’ general participation in various surveys in general appears to minimize this self selection for a particular topic (t = 5.60). Presumably the largest self-selection factors for the mall intercept sample were the inclination to visit a mall and the ability to spare the time.

Overall, in achieving the four goals of a successful survey implementation mentioned in Section 4, the two modes that first contact respondents by phone (phone-mail and central location) tend to have difficulties in the ability to contact potential respondents, as those who agree to participate are older, wealthier, and more educated than the target population overall. The mall intercept mode also has difficulties, as it reaches a greater number of younger, less wealthy, less educated, and unmarried respondents. The Internet panel does not have such difficulties in terms of demographics, since the characteristics of potential respondents are known in advance of any particular survey invitation. The two modes using telephone contact also perform poorly on minimizing the influence of potential respondents with a particular interest in the survey topic, as measured by their significantly higher use of environmental amenities than the other modes. The mall intercept and Internet panel did better by that measure.

## 6. Valuation Differences among Modes

Because each mode generated samples with different demographic characteristics, one would expect differences, to the extent that those water quality valuations are driven by demographic factors. We used the two-tailed Tobit regression analysis shown in Table 3 to generate estimates of the water quality valuations that account for demographic differences and censoring effects. The two-tailed Tobit approach is appropriate because some of the respondents reached the lower left or right side of the decision tree in Figure 3.

Their actual value could be much lower than the assigned low value or much higher than the assigned high value. The use of Tobit regressions accounts for that indeterminacy. As the results in Table 3 indicate, relative to the Internet panel, phone-mail and mall intercept modes generate greater valuations of improved water quality after accounting for demographic characteristics. Despite the small sample sizes for these modes relative to the Internet panel group, the differences are significant at the 1% level. The estimates in Table 3 also demonstrate the effects of demographic differences on values. As the table shows, values for regional water quality amenities differ based upon interest in the environment (as measured by membership in an environmental organization), direct use of the good through visits, household income, education, age, and minority status. However, even after accounting for those factors as well as differences in starting points and baseline water quality between surveys, the phone-mail and mall intercept samples still have substantially higher values than the Internet panel or central location administration.

Table 4 shows how these differences are reflected in estimates of the water quality values. First, each mode was assigned the average demographic values of everyone in the complete sample of all surveys regardless of mode. Therefore, even though, for example, the central location sample was much older in practice and started with a lower initial cost-quality tradeoff ratio, they are assigned the average age and starting point ratio for this calculation. These adjustments to all measured factors except survey mode reveal the extent to which the survey mode affects estimated values of regional water quality. Compared to the Internet panel, the phone-mail sample had estimated values 71% higher, mall intercept 37% higher, and the central location sample 6% lower. While there was a substantial time difference between surveys (1997–2004), it is unlikely the results are due to changing tastes over time on the part of the public, as the differences are dramatic and the two lowest valuations are the first and last mode investigated over the time period. However, the amount of time across surveys does allow for the possibility that there could be unaccounted for exogenous effects.

While the regression accounts for systematic differences between the modes, there appear to be differences in participation not reflected in the demographic, visitation, or survey text differences that increase valuations for phone-mail and mall intercept surveys. Since all the surveys were implemented on computers, the differences likely arise largely from self selection and the physical environment where computers were used. This latter factor is discussed below in an examination of the inconsistency of responses.

## 7. Level of Inconsistent Responses among Modes

Aside from substantial differences in valuation of water quality, the four different survey modes differed in terms of how often respondents passed the consistency test. A consistency test is important because it is a measure of the extent to which respondents understand and are attentive to the survey task, and accordingly minimize irrational or protest responses.

For this survey, a respondent was treated as being inconsistent if the respondent continued to choose the lower cost or the higher quality region even when it became a dominated option. For respondents on either extreme side of the decision tree, the survey provided a dominated choice, where the region they had consistently chosen was made to be clearly worse than the other option. This choice either had the same water quality in both regions with one having a higher cost of living (for respondents who had been choosing higher quality) or had the same cost of living with one having a higher water quality (for respondents who had been choosing lower cost of living). If the respondent then chose the dominated region, the respondent was informed about that inconsistency. If the respondent still persisted in that choice, that respondent was deemed inconsistent and not included in the regular analysis.

Table 5 shows the differences in the percentage of inconsistent respondents for each survey mode. While an average of 5.3% of respondents were inconsistent across all the surveys, that rate ranged from a low of 3.5% for the phone-mail sample to a high of 12.9% for the mall intercept sample. In terms of difference in proportion tests, the Internet panel has significantly fewer inconsistent respondents than the surveys using other modes, as well as significantly fewer insignificant responses at the high value portion of the decision tree compared to the other modes (z statistics of 5.03 and 4.91 respectively). The mall intercept sample was worse on inconsistency overall, as well as inconsistency at both the high and low value questions (z statistics of 6.08, 5.88, and 2.0 respectively). While phone-mail had the lowest nominal level of inconsistency, that difference was not significantly lower than the overall sample or than the Internet panel.

The demographic characteristics of inconsistent respondents are reflected in the probit regressions in Table 6 for whether the respondent is inconsistent, where the probit coefficients have been transformed to equal marginal effects. The omitted survey category group serving as the reference point for these estimates is the Internet panel sample. The differences by income and by whether the respondent had visited a lake or river suggest that not all inconsistency may be due to inattentiveness or confusion. Some of the respondents classified as being inconsistent may be registering a sort of protest or merely an insistence on the direction of previous answers. After accounting for demographic and starting point differences, the mall intercept sample is more inconsistent overall and is more prone to being inconsistent if the respondent has a high valuation relative to the Internet panel sample, and the phone-mail sample is less inconsistent at the high valuations.

The relatively large percent of inconsistent respondents for the mall intercept sample is cause for concern. These respondents were invited to interrupt their shopping to take a survey in a room within a mall. If these respondents were more likely to be uncomfortable, impatient, rushed, or in some way affected by participating in the survey due to their attention being focused elsewhere, then this survey mode may be inappropriate, particularly for more complicated surveys.

The lower levels of inconsistency in the Internet panel and the phone-mail samples illuminate this possibility. For these samples, respondents generally completed the survey in their own homes at a convenient time of their choosing. This comfort may have resulted in better attention, less hurriedness, and therefore more thoughtful and fewer inconsistent responses.

Since Internet panel and phone-mail respondents were generally able to complete the survey in their own homes, the lower rate of inconsistency is understandable. The central location and mall intercept respondents may have felt hurried or simply not completely comfortable in a foreign environment. For their parts, central location respondents may have outperformed mall intercept since the former committed to a specific trip to participate. Mall intercept respondents were merely convinced to participate during an already planned outing, and thereby may not have given the survey as much attention as the central location group. In terms of the last factor in a successful survey implementation from Section 4, the Internet panel and phone-mail sample perform best, while the central location and mall intercept modes have difficulty in ensuring a comfortable environment in which to take a survey.

## 8. Non-Response Characteristics

Unfortunately, it is difficult to precisely identify effects of survey modes when non-response is involved. Except for the Internet panel, little information is available about those who declined to participate. The Internet panel sample provides insight about the characteristics of those who declined to participate in that survey. While offering little information about people who refuse to be part of the panel, it is instructive to identify those panel members who declined the invitation to participate in this particular survey. Knowledge Networks’ Internet panel has a broad set of basic demographic characteristics that is collected independently of our survey. Table 7 compares demographic information for those who completed the survey with those on the panel who declined the invitation to participate in this survey, and Table 8 presents a probit regression reporting significant marginal effects of those characteristics on the probability of participation in the survey. In general, older and more educated invitees agreed to complete the survey, while those at the top income category and those in defined minority groups were less likely to agree to take the survey.

Comparing Table 7 with Table 2, phone-mail mode most closely tracks these results. Phone-mail had more educated, older, fewer black, and fewer Hispanic respondents, all significant predictors of participation in the probit regression results in Table 8. In addition, the phone-mail respondents were more likely to be married and had higher incomes than the Internet panel respondents, both of which were seen in Table 6 but are not statistically significant in the probit estimates.

The central location mode only corresponded with non-response expectations in terms of years of education and Hispanic ethnicity. Otherwise, these respondents were somewhat younger, possibly due to the travel requirement to participate at a central location.

The mall intercept mode also accorded with expectations in terms of education and Hispanic ethnicity. However, these respondents were younger, more likely to be black, had lower incomes, and were less likely to be married than respondents in the Internet panel. This could be due to the demographic characteristics of shoppers at the mall where the respondents were recruited, as well as mall shoppers who had enough time available to complete a survey.

So, generally, each mode’s demographic makeup corresponds, to some extent, to the factors seen in the Internet panel that affected participation, but each also has participation affected by features of its own sampling characteristics. The consistent effects of such factors on survey participation are cause for concern in terms of achieving a nationally representative sample and provide a reason to use modes that are less affected by self selection.

## 9. Conclusions

We examined two ways in which four survey recruiting modes might influence the valuation outcomes of an environmental good. First, we showed that survey mode influences the characteristics of those who choose to respond to the survey. Second, we demonstrated that the different survey modes affect whether the survey elicits their preferences accurately.

There are significant and large differences in the valuation of an environmental good across recruitment modes of survey administration. These differences persist even when demographic and survey question differences are accounted for, and are most likely associated with self selection by respondents who are interested in the topic when recruited by phone. This effect seems to be smallest for the probability sampled Internet panel, where respondents agree in advance to take a number of surveys on a range of topics.

Further research might investigate other modes, such as door-to-door surveys, to compare the trade-off in inconsistency associated with location comfort against time constraint, as well as non-response characteristics. In addition, interest in environmental goods and issues could be assessed in surveys on unrelated topics to determine the extent of self selection by topic even among the Internet panel. Such an assessment would serve to determine a baseline to measure how the level of interest affects participation.

What features are best suited for assembling a representative and unbiased sample? A survey mode for a study should have characteristics that lead to as representative a sample as feasible, including factors that maximize the ability to project the sample to a well-defined universe, minimizing self selection in participation by those with particular interest in or disinclination towards the survey topic, minimizing and compensating for the costs incurred by respondents to complete the survey task, and maximizing the ability of the respondent to be comfortable in the manner and location in which the survey is administered.

Table 9 evaluates the four modes based on these criteria. Based on this summary scorecard, the Internet panel performs as well or better on all four criteria. The availability of demographic information among panelists gives the Internet panel the ability to produce a sample that is nationally representative in terms of the most common demographic characteristics. While the fact that panelists agree in advance to take surveys on a variety of topics does not eliminate the possibility of self selection, it minimizes this effect better than any of the other modes where potential respondents are informed in advance of the survey topic. The Internet panel also minimizes time and effort costs associated with completing the survey task by allowing respondents to complete the survey in their own home and at a time of their choosing.

Other survey modes perform well with respect to one or more of these factors, but none perform overall as well as the Internet panel. For instance, while phone-mail allows most respondents to complete the survey in their home at a convenient time, this survey mode requires additional software or requires non-computer users to take the survey elsewhere. Mall intercept minimizes travel time since respondents are already at the survey location, but their lack of consistency may derive from feeling rushed by the interruption of their trip or lack of comfort in an unfamiliar survey environment.

Inconsistency in responses to survey questions can also indicate problems with a particular survey mode, as it indicates lack of attention and thoughtfulness toward the survey task. The mall intercept sample was most often associated with inconsistent responses in the survey, perhaps due to rushed or uncomfortable respondents. Phone-mail and the Internet panel modes, where respondents were most able to complete the survey at a time and place of their choosing, had the least such inconsistency.

Non-response, as measured using invited Internet panelists who declined to participate, is associated with a variety of demographic characteristics, and those characteristics are reflected to some extent in the make-up of each of the other survey modes. Those differences were most similar to the phone-mail mode, where respondents were reached by random digit dialing as in the original recruitment of the Internet panel. Other modes had some such similarities, which were somewhat confounded by characteristics particular to each survey mode.

In general, it is clear that the choice of survey recruitment mode can affect the estimation of the value of an environmental good. The Internet panel seems to minimize such effects among survey modes investigated in this research.

## Figures and Tables

**Table 1.** Characteristics and timing of the survey modes.

**Survey Mode**	**Date**	**Interviews**	**% of Total**
Total Number of Interviews	1997–2004	5,122	100%
Central Location, Research Triangle Park, NC	August 1997	106	2%
Mall Intercept, Cary, Charlotte, Co. Springs, Denver	January 1998	303	6%
National Phone-mail 1	September 1999	33	1%
National Phone-mail 2	June 2000	53	1%
Internet Panel Pretest	December 2001	383	7%
Internet Panel Round 1	October 2002	184	4%
Internet Panel Round 2	February 2003	406	8%
Internet Panel Round 3	April 2003	580	11%
Internet Panel Round 4	April 2004	549	11%
Internet Panel Round 5	August 2004	516	10%
Internet Panel Round 6	October 2004	2,009	39%

**Table 2.** Comparison of sample to the national adult U.S. population ^a^.

**Demographic Variable**	**US Adult Population 2000**	**Full Sample (n = 5,122)**	**Internet Panel (n = 4,627)**	**Phone–Mail (n = 86)**	**Mall Intercept (n = 303)**	**Central Location (n = 106)**
***Gender***						
Male	48.1%	50.9%	50.9%	58.1%	49.8%	47.2%
Female	51.9%	49.1%	49.1%	41.9%	50.2%	52.8%
***Age***						
18–24 years old	13.0%	14.1%	13.0%	3.5%	35.3%	7.6%
25–34 years old	18.3%	20.0%	19.7%	5.8%	25.7%	27.4%
35–44 years old	21.9%	19.6%	19.7%	23.3%	16.5%	25.5%
45–54 years old	18.1%	18.8%	18.8%	30.2%	13.9%	21.7%
55–64 years old	11.7%	11.8%	12.3%	22.1%	3.3%	8.5%
65–74 years old	8.9%	11.2%	11.6%	15.1%	5.3%	9.4%
75 years or older	8.1%	4.5%	5.0%	0%	0%	0%
***Age, Mean***		44.42	44.90	50.86	35.15	42.55
***Educational Attainment***						
Less than high school diploma	15.8%	16.7%	17.8%	2.3%	9.2%	1.9%
High school diploma or higher	58.5%	59.0%	59.9%	47.7%	61.4%	22.6%
Bachelor’s degree or higher	25.6%	24.3%	22.3%	50.0%	29.4%	75.5%
***Years of Education, Mean***		13.35	13.21	15.10	14.06	16.19
***Race/Ethnicity***						
White	83.0%	79.1%	79.5%	89.5%	77.6%	81.2%
Black/African-American	11.9%	12.9%	13.0%	3.5%	15.5%	11.3%
Other Race	5.0%	7.6%	7.6%	7.0%	6.9%	7.5%
Hispanic	9.9%	10.0%	10.6%	3.5%	5.9%	1.9%
***Marital Status***						
Not married	40.5%	43.4%	42.8%	26.7%	59.7%	36.8%
Married	59.5%	56.6%	57.2%	73.3%	40.3%	63.2%
***Household Income***						
Less than $15,000	15.9%	14.5%	14.4%	3.5%	20.5%	8.5%
$15,000 to $24,999	13.4 %	13.3%	11.5%	12.8%	39.9%	14.2%
$25,000 to $34,999	12.5 %	11.5%	12.8%	0%	0%	0%
$35,000 to $49,999	15.5 %	20.0%	19.4%	36.1%	22.1%	30.2%
$50,000 to $74,999	18.9 %	16.7%	18.5%	0%	0 %	0%
$75,000 to $99,999	10.4 %	15.0%	14.2%	34.9%	13.2%	38.7%
$100,000 or more	13.4 %	9.0%	9.3%	12.8%	4.3%	8.5%
***Household Income, Mean***		$49,784	$50,538	$56,065	$33,735	$53,773
***Member of Environmental Organization***		5.7%	5.3%	6.0%	7.6%	18.4%
***Visited Lake or River, Last 12 Months***		69.0%	67.7%	90.4%	75.4%	88.8%

**Table 3.** Censored-normal regression of log of regional water quality value ^a^.

**Log (Regional Water Quality Value)**	**Coefficient**	**Standard Error**
Survey Mode, Phone–Mail	0.5390 ***	0.1485
Survey Mode, Mall Intercept	0.3134 ***	0.0877
Survey Mode, Central Location	−0.0643	0.1392
Starting Ratio	0.0308 ***	0.0039
Baseline Quality	−0.0061 ***	0.0014
Member of Environmental Org.	0.4098 ***	0.0781
Visited Lake or River, Last 12 Months	0.2160 ***	0.0397
Log (Income)	0.1028 ***	0.0228
Top Income Category	0.2282 *	0.1218
Missing Data, Income	−0.1514	0.2174
Years of Education	0.0419 ***	0.0073
Age	0.0067 ***	0.0011
Black	−0.1313 **	0.0557
Other	−0.0705	0.0687
Hispanic	0.0695	0.0608
Female	−0.0340	0.0356
Married	0.0567	0.0385
Northeast	0.0525	0.0573
South	0.0067	0.0488
West	−0.0155	0.0554
Intercept	0.3791	0.2570
Observations	4,851	
LR chi2(18)	306.74	
Prob > chi2	0.0000	
Pseudo R2	0.0204	
Log likelihood	−7,362.5349	

a Notes:

* significant at 10%;

** significant at 5%;

*** significant at 1%; 406 observations were low censored, 569 observations were high censored.

**Table 4.** Estimated water quality values, censoring of extreme values, and starting points.

**Variable**	**Full Sample**	**Central Location**	**Mall Intercept**	**Phone-mail**	**Internet Panel**
Observations	4,851	98	264	83	4,406
*Using Full Sample Average Demographics*					
Estimated Regional Water Value (Log)	$32.10	$29.36	$42.83	$53.67	$31.31
Difference from Internet Panel (Log)		−6%	+37%	+71%	
Censored High	11.7%	15.3%	23.9%	36.1%	10.5%
Censored Low	8.4%	2.0%	5.3%	1.2%	8.8%
Starting Ratio	$15.49	$4	$10	$10	$16.18
Baseline Quality	53.65%	50%	49.9%	50%	54.03%

**Table 5.** Percent inconsistent responses by survey mode.

**Percent Inconsistent**	**N**	**Total**	**Inconsistent at High Value**	**Inconsistent at Low Value**
Survey Mode, Internet Panel	4,627	4.78%	3.28%	1.49%
Survey Mode, Phone–Mail	86	3.49%	1.16%	2.33%
Survey Mode, Mall Intercept	303	12.87%	9.90%	2.97%
Survey Mode, Central Location	106	7.54%	6.60%	0.94%

**Table 6.** Probit regressions predicting inconsistency using demographic characteristics ^a^.

	**Inconsistent (Any)**	**Inconsistent (High Value)**	**Inconsistent (Low Value)**
Survey Mode, Central Location	0.0355 (0.0320)	0.0200 (0.0238)	0.0085 (0.0211)
Survey Mode, Mall Intercept	0.0655 *** (0.0211)	0.0485 *** (0.0180)	0.0166 (0.0114)
Survey Mode, Phone–Mail	−0.0126 (0.0218)	**−**0.0249 ** (0.0103)	0.0211 (0.0242)
Visited Lake or River, Last 12 Months	−0.0161 ** (0.0071)	**−**0.0069 (0.0058)	**−**0.0080 ** (0.0038)
Log (Income)	−0.0036 (0.0038)	0.0029 (0.0033)	**−**0.0045 *** (0.0017)
Years of Education	−0.0014 (0.0012)	0.0003 (0.0010)	**−**0.0017 *** (0.0006)
Female	−0.0136 ** (0.0060)	**−**0.0062 (0.0050)	**−**0.0068 ** (0.0030)
Northeast	0.0066 (0.0111)	0.0033 (0.0092)	0.0025 (0.0056)
South	0.0054 (0.0090)	0.0034 (0.0076)	0.0019 (0.0045)
West	0.0189 * (0.0110)	0.0157 * (0.0094)	0.0026 (0.0053)
Observations	5,122	5,122	5,122
LR chi2(19)	58.65	50.76	41.06
Pseudo R2	0.0277	0.0312	0.0493
Log likelihood	**−**1,030.8938	**−**786.96557	**−**395.7234

a Notes: Coefficients have been transformed to equal marginal effects; Standard errors in parentheses,

* significant at 10%;

** significant at 5%;

*** significant at 1%; Not shown here, not significant, but included in the model are starting ratio, baseline quality, member of environmental organization, top income category, income data missing, age, black, other race, Hispanic, and married.

**Table 7.** Non-response characteristics in Internet panel.

**Variable**	**Completed Survey**	**Declined Invitation**
Income	$51,671	$50,862
Top Income Category	1.9%	3.4%
Years of Education	13.17	12.82
Age (Years)	44.70	37.37
Black	13.4%	20.5%
Other	6.4%	7.5%
Hispanic	10.6%	14.8%
Female	49.0%	50.5%
Married	56.4%	49.0%
Northeast	18.5%	18.8%
South	36.2%	38.1%
West	21.6%	22.0%
N	4,249	1,393

**Table 8.** Probit regressions predicting non-response in Internet panel ^a^.

**Accepted Invitation to Participate in Survey**	**Coefficient**	**Std. Err.**
Log (Income)	0.0025	0.0067
Top Income Category	−0.1697 ***	0.0465
Years of Education	0.0100 ***	0.0023
Age	0.0050 ***	0.0004
Black	−0.1107 ***	0.0181
Other	−0.0490 **	0.0250
Hispanic	−0.0805 ***	0.0196
Female	−0.0084	0.0115
Married	0.0047	0.0123
Northeast	−0.0225	0.0186
South	−0.0209	0.0157
West	−0.0208	0.0181
Observations	5,642	
LR chi2(11)	310.13	
Pseudo R2	0.0492	
Log likelihood	−2,998.2614	

a Notes: Coefficients have been transformed to equal marginal effects;

* significant at 10%;

** significant at 5%;

*** significant at 1%.

**Table 9.** Participation factors and the performance among survey modes.

	**Internet Panel**	**Phone-Mail**	**Mall Intercept**	**Central Location**
	**Good.**	**Fair.**	**Poor.**	**Fair.**
Ability of investigators to project to a well-defined universe	Though panelists must be recruited to the panel by phone, members are generally willing to complete surveys, and the characteristics of invitees are available.	Households are difficult to reach by phone, and those who can more easily be reached may have different demographic characteristics than the US adult population.	Invitees are already present, but the demographics of mall visitors may be different than US population.	Households are difficult to reach by phone, and those who can more easily be reached have different demographic characteristics than the US adult population.

	**Good.**	**Poor.**	**Poor.**	**Poor.**
Self selection by respondents who are positive toward the topic	Since panelists already agree to take a variety of surveys, self selection by topic is lessened.	Phoned invitees can opt in if particularly interested or opt out if they do not feel they are knowledgeable about the topic.	Invited shoppers can opt in if particularly interested or opt out if they do not feel they are knowledgeable about the topic.	Phoned invitees can opt in if particularly interested or opt out if they do not feel they are knowledgeable about the topic.

	**Excellent.**	**Good.**	**Fair.**	**Poor.**
Total time and effort costs for respondents to complete the survey	Invitation to participate sent by e-mail, survey completed in the home.	Invitation by phone, survey disk by mail, survey completed by most respondents at home, and completed survey returned by mail.	Invited shoppers are already at the survey location, but must interrupt an activity already in progress.	Phoned invitees must travel to survey location.

	**Excellent.**	**Excellent or Good.**	**Poor.**	**Poor.**
Ability of respondent to be comfortable in the location where the survey is completed	Respondents complete the survey at a time of their convenience in their own home.	Most respondents complete the survey at a time of their convenience in their own home. Some might travel to a location with an available computer to complete the survey.	Respondents complete the survey in an unfamiliar location at the time of the shopping trip.	Respondents complete the survey in an unfamiliar location at a scheduled time when the central location is open.
		All respondents must return the materials by mail.		
